# Multimodal experimental data for tandem gas metal arc welding of thick steel plates with advanced pulsed and short-circuiting process modes

**DOI:** 10.1016/j.dib.2026.113329

**Published:** 2026-10-10

**Authors:** Çağtay Fabry, Prashant Kumar Chaurasia, Andreas Pittner, Michael Rethmeier

**Affiliations:** a Bundesanstalt für Materialforschung und –prüfung (BAM), Berlin, 12205, Germany; b University West, Trollhättan, 461 86, Sweden; c Technische Universität Berlin, Berlin, 10623, Germany; d Fraunhofer IPK, Berlin, 10587, Germany

**Keywords:** Tandem gas metal arc welding, Process monitoring, Surface scanning, Electrical deposition efficiency, Power consumption, Steel, Pulsed mode, Controlled short-circuiting mode

## Abstract

High power tandem gas metal arc welding (T-GMAW) enables increased productivity in thick plate applications by supplying two wires to a single melt pool. Besides increasing the deposition rate, T-GMAW has previously been demonstrated to offer improved energy efficiency and a reduced environmental footprint compared to single wire process variants. However, due to the complex interaction of both arc processes, achieving stable and reliable welding conditions has historically been challenging, especially at high deposition rates. Introduction of advanced process mode variants for pulsed and controlled short-circuiting transfer modes have increased the stability of the T-GMAW process and widened the field of applications. Since these new technologies are emerging and availability of publications and datasets in T-GMAW is limited – especially with modern equipment and process modes – we want to present an initial comprehensive multimodal T-GMAW dataset covering a variety of process mode combinations and deposition rates. A common multi-layer V-Groove joint design is chosen to reflect real world thick plate applications. The dataset covers high resolution time series recordings of the welding process parameters, its arc process signatures, power and material consumptions and the exact movement of the welding torch along the groove in a weaving pattern. Furthermore, detailed 3D measurements of the weld seam surface are included for each layer together with high dynamic range video recordings of the T-GMAW process. The dataset enables researchers to analyse the process in a variety of ways. Possible applications could include the calculations of heat input and process efficiency dependent on the chosen process parameters or evaluation of the process signatures in relation to the groove geometry and weaving pattern for arc sensing. The provided video recordings also allow estimations of the melt pool size and shape which are widely used as inputs for welding simulations. Researchers interested in life-cycle inventory development can compare the advanced T-GMAW process to other joining and manufacturing processes based on the included data.

Specifications Table

**Table:** 

Subject	Engineering & Materials science
Specific subject area	Process monitoring for tandem gas metal arc welding. Comparison of arc process modes, deposition rates, power consumption and bead shape.
Type of data	Raw Time series (NetCDF/HDF5 format) 3D point cloud (NetCDF/HDF5 format) Video (.avi format) Supporting materials (experiment list and overview, example code file)
Data collection	A total of five mild steel specimen of 30 mm thickness were prepared with 50° V-Groove and 1–2 mm root gap. Two Fronius TPS-500i power sources in tandem configuration were used to weld 5 filler layers with two 1.2 mm ER70S-6 wires under 82%Ar-18%CO_2_ shielding gas for each specimen. A single bead-per-layer welding strategy was implemented at varying welding travel speeds with lateral weaving. Time series for process parameters, arc welding signatures, power and shielding gas consumption were collected using a TwinCAT3 control system. 3D surface data before and after each weld pass was collected using a Micro-Epsilon scanCONTROL 3010–100 sensor.
Data source location	Bundesanstalt für Materialforschung und -prüfung (BAM), Berlin, Germany
Data accessibility	Repository name: Zenodo Data identification number: https://doi.org/10.5281/zenodo.21824586 Direct URL to data: https://zenodo.org/records/21824586
Related research article	P. K. Chaurasia, Ç. Fabry, A. Pittner, A. De, M. Rethmeier, “Tandem Gas Metal Arc Welding of Thick Steel Plates: Progress in Productivity and Properties using Advanced Process Modes”, *Science and Technology of Welding and Joining,* 2026, https://doi.org/10.1177/13621718261472548 [1]

## 1 Value of the Data

• The dataset [2] offers valuable insight into an advanced Tandem Gas Metal Arc Welding joining process in a conventional V-Groove joint, serving as an example for a high productivity arc welding process for thick steel plate applications. Multiple individual multilayer experiments are included for combination of different advanced process modes and deposition rates for the leading and trailing wire. Each experiment contains measurements for five T—GMAW filler layers deposited after an initial root pass in single wire pulsed welding. The dataset covers pulsed operation using the proprietary pulse multi control (PMC) process and controlled short-circuiting operation in the proprietary cold metal transfer (CMT) process. The T—GMAW scenarios covered are PMC + PMC operation for the highest possible deposition rate of up to 35 m/min combined wire feed rate, PMC + CMT for high deposition rates at improved electrical deposition efficiency up to 32 m/min combined wire feed rate and CMT + CMT for the lowest possible heat input and highest electrical deposition efficiency at 24 m/min combined wire feed rate. The dataset further includes PMC+PMC and PMC+CMT experiments at the same combined wire feed rate of 24 m/min.
• The dataset combines multimodal data in a common spatiotemporal reference frame and includes high resolution time series recordings of the arc welding process signatures (welding current and voltage) that are synchronized to the time series recordings of the welding path and process parameters. Point cloud data representing the finished weld bead surface of each individual layer is given in the same reference frame as the welding path. The dataset further includes the recorded energy and materials consumption relevant to the welding process.
• The dataset serves as a valuable reference of a not yet widespread manufacturing technology that allows researchers to compare it to results for more conventional single wire arc welding applications or other joining technologies for thick plate applications such as laser beam welding regarding heat input, materials consumption and productivity.
• Researchers can analyze the data to conduct further analysis in the process behavior and stability of advanced pulsed and controlled short-circuiting modes at high deposition rates and with complex weaving motion in an application example. These could include initial investigations into arc sensing based control or sensitivity studies. The dataset can also be used as supplemental validation or calibration data for welding simulations or machine learning applications regarding process behavior, weld bead forming or thermal simulations. For a broader scope, recorded materials consumption can support life-cycle inventory development and contribute welding process related inputs for further use in Life Cycle Assessments.

## 2 Background

Tandem gas metal arc welding (T-GMAW) has demonstrated to be a viable manufacturing technology to improve productivity, efficiency and sustainability for welding thin and thick steel applications compared to conventional single wire processes [3,4]. Due to the complex interactions of the welding process of both wires feeding into the joint melt pool, ensuring stable process conditions has been an ongoing area of research [5]. While previous research publications covering the process behaviour or efficiency have been published [6], datasets allowing further analysis and comparison are not yet publicly available. In recent years, technological advancements in controlling arc welding processes have been introduced to T-GMAW, enabling advanced pulsed and controlled short-circuiting operation to increase process stability and efficiency further. We want to share an initial comprehensive multimodal dataset covering the behaviour of modern T-GMAW operation for joining of thick steel applications when using pulsed and controlled short-circuiting modes. While the high-resolution transient recordings allow detailed analysis of the arc welding process, additional surface and power consumption measurements are included to broaden the possible use cases of the dataset. The 50° V-Groove joint design and welding materials have been chosen to allow direct comparison to previous studies regarding environmental impact [4].

## 3 Data Description

This article describes the dataset [2] collected during welding experiments using a T-GMAW process in a 50° V-Groove joint for 30 mm thick steel plates. Experiments were carried out by applying advanced pulsed (Fronius PMC) and controlled short-circuiting (Fronius CMT) process modes. The dataset covers five distinct experiments using a variety of process mode selection and deposition rates combinations. For each experiment, six individual welding layers are recorded. For all experiments, the first layer is welded with a single wire GMAW process using the same PMC process conditions to provide the initial root pass. The following five filler layers are welded in the dual wire T—GMAW process configurations described below. Measurements for the single wire root passes are included in the dataset for completeness and to allow reproduction as well as the direct comparison of the data with previously published results [4]. As such, the single wire experiments should not be included in analysis or comparison strictly limited to T-GMAW results. A detailed overview of all individual recordings with the most relevant welding parameters is included in the dataset as **experiment_list.csv** described in Table 1.

**Table 1:** column description of dataset overview file **experiment_list.csv.**

column name	unit	description
wid	-	numeric identifier for the experiment (one of [615, 617, 630, 631, 632])
layer	-	weldment layer number of the record (range 1–6)
track	-	track number of the record (fixed at 1)
config	-	welding configuration used (single: single wire GMAW process using the leading power source, tandem: leading and trailing wire T-GMAW process)
wts	mm / s	The programmed welding travel speed in direction of the groove (projected onto the x-direction of the welding reference frame)
mode_leading	-	the selected leading wire process mode (PMC or CMT)
mode_trailing	-	the selected trailing wire process mode (PMC or CMT)
wfs_command_leading	m / min	the selected leading wire target wire feed rate
wfs_command_trailing	m / min	the selected trailing wire target wire feed rate
wfs_command_combined	m / min	the combined target wire feed rate for leading and trailing wires
weave_amp	mm	the programmed lateral weaving amplitude in y-direction of the welding reference frame
weave_freq	Hz	the programmed weaving frequency of the robot motion
t85	s	the measured t_8/5_ cooling time from 800 °C to 500 °C (not available for all records, see LIMITATIONS)
stem	-	the general file prefix pattern for the recording
scan_file	-	filename of 3D surface scan before welding
robot_file	-	filename of robot time series data
process_file	-	filename of process time series data
ps_leading_file	-	filename of leading wire power source settings and measurement time series data
ps_trailing_file	-	filename of trailing wire power source settings and measurement time series data (not present for initial root pass layers with config “single”)
video_file	-	filename of HDR video recording

The dataset contains a single zip file for each layer with all recordings which includes the individual time series data files, the 3D surface data file and video file as listed in Table 1. Fig. 1 shows the directory tree of the dataset with the contents of the zip file for recording WID 615 – Layer 2 for reference. The file naming schema is based on the unique numeric experiment identifier *WID{wid}* and the welding layer *L{layer}* for each recording, for example the prefix *WID630_L3* would describe files related to the third layer recording of experiment 630. Since the initial first layer is welded in single wire configuration using the leading wire only, no trailing power source measurements are provided for the *L1* layers.

**Fig. 1:**
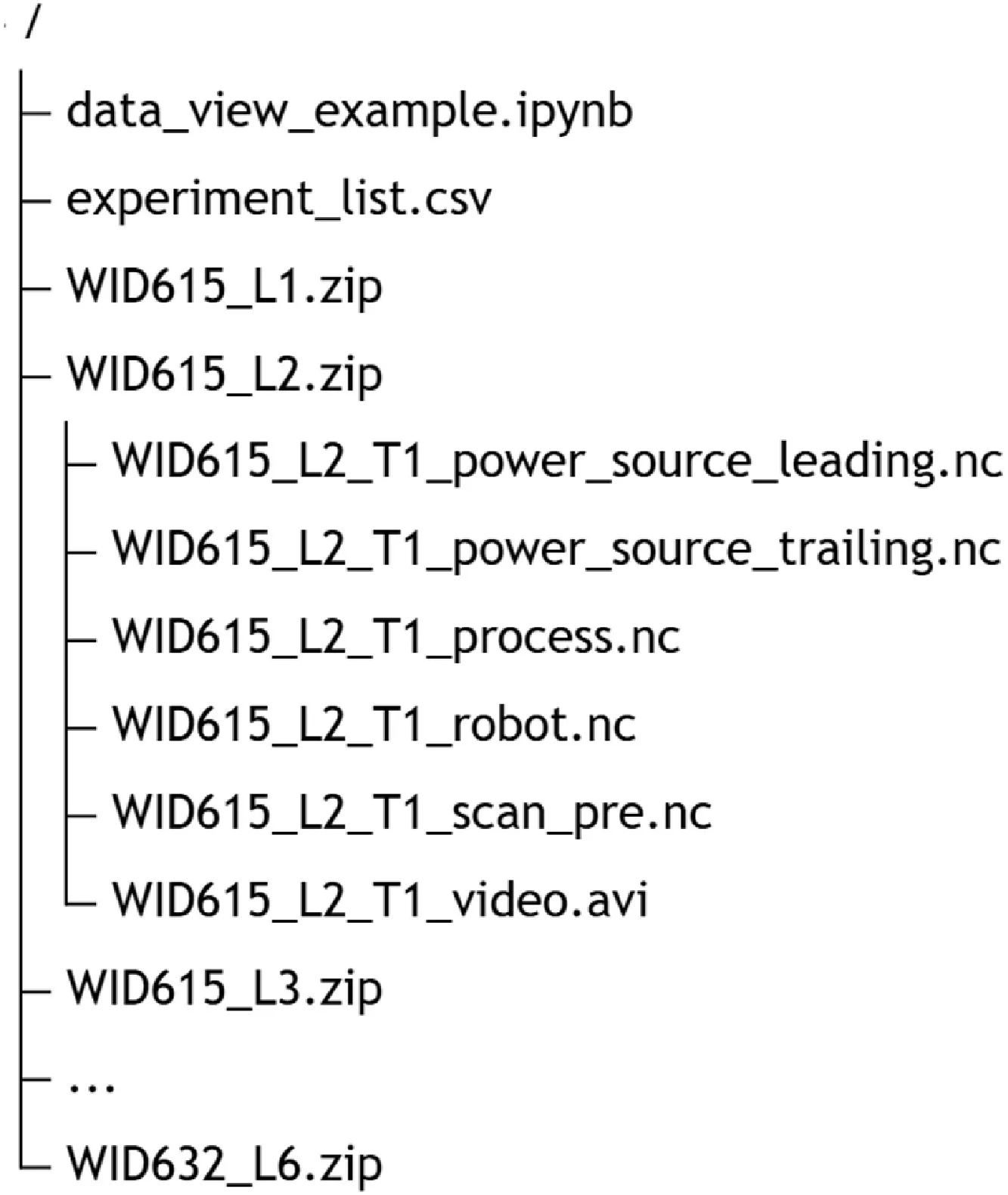
File structure of the dataset and exemplary contents of zip file for a single recording.

### 3.1 Time series data

The time series data files are stored as HDF5 files in the NetCDF4 format. They have been created using the xarray Python library, which is the preferred way to read the data. Each time series file has a data variable *time* that is used as the primary coordinate for all other data variables and given as a UTC timestamp. All timestamps across all files reference the same master clock of the control system described in the measurement setup section below (see Fig. 6). The minimum sampling rates vary by file depending on the required resolution and source of data. For the *robot_file* the sampling rate is 1 ms, for the *process_file* it is 0.1 ms and for the power source files *ps_leading_file* and *ps_trailing_file* it is 100 ms. The sampling interval for each data variable is based on the shortest interval provided by the corresponding data source. Robot positions are transferred from the robot to the control system in 8 ms intervals and OPC-UA communication of the between the power source and control system is limited to 100 ms sampling intervals. All variables are described with units and individual sampling rate in Table 2. In total, the dataset contains 1333.665 s of time series data for welding with T-GMAW experiments across all *process_file*s.

**Table 2:** List and description of data variables for time series measurements (further internal variables recorded from the power source are included in the dataset but omitted from this list for brevity).

file type (see Table 1)	data variable name	description	unit	Interval in ms
robot_file	tcp_coords	measured TCP xyz-coordinate in welding reference frame	mm	8
	tcp_angles	measured TCP orientation as xyz euler angles in welding reference frame	degree	8
	tcp_tool_number	current active tool number	-	8
	gas_flow_leading	measured shielding gas flow leading power source at gas supply	l/min	1
	gas_flow_trailing	measured shielding gas flow trailing power source at gas supply	l/min	1
	power_leading	measured power draw of leading power source from wall plug	W	1
	power_trailing	measured power draw of trailing power source from wall plug	W	1
	power_robot	measured power draw of robot system from wall plug	W	1
	arc_on	confirmation of active welding process from power source	-	1
	thermo_1	temperature measured by thermocouple placed on workpiece surface	°C	1
	thermo_2	temperature measured by thermocouple placed in weld pool	°C	1
process_file	current_leading	welding current measurement of leading wire	A	0.1
	voltage_leading	welding voltage measurement of leading wire	V	0.1
	current_trailing	welding current measurement of trailing wire	A	0.1
	voltage_trailing	welding voltage measurement of trailing wire	V	0.1
	video_frame	HDR video image capture trigger (positive edge indicates frame capture start)	-	0.1
	video_recording	timeframe of video recording	-	0.1
ps_leading_file & ps_trailing_file	PROCESS_MAINPHASE	trigger signal indicating the running welding process	-	100
	WFS_COMMANDVALUE	target wire feed rate	m/min	100
	ACTUAL_WFS	measured wire feed rate	m/min	100
	ACTUAL_CURRENT	measured welding current	A	100
	ACTUAL_VOLTAGE	measured welding voltage	V	100
	ACTUAL_POWER	measured arc energy	W	100
	ACTUAL_GASFLOW	measured gas flow		100

Fig. 2 shows selected exemplary time series data for recording WID 632 – Layer 3. The recordings for wire feed rate and welding current and voltage are only active during the welding process while the power draw and robot TCP recording covers the complete welding sequence including 3D measurements. Fig. 3 shows a detailed view of the transient welding current and voltage recordings for both wires over a selected timespan of 0.1 s chosen from Fig. 2, highlighting the high sampling frequency and capture of the dynamic welding process signatures. It also shows exemplary releases of the HDR video frame trigger which is activated on positive edge detection of the trigger signal.

**Fig. 2:**
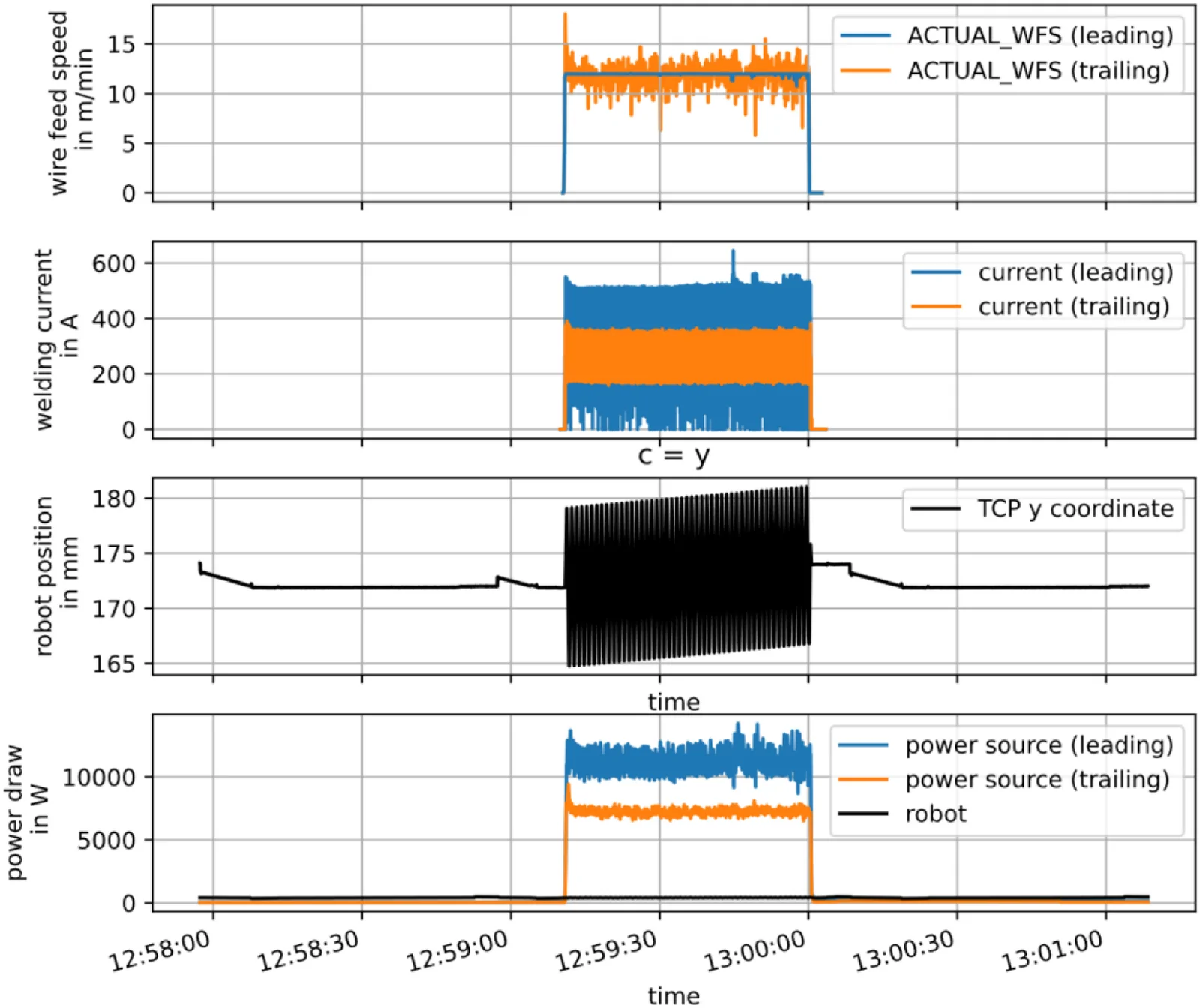
exemplary overview of measured wire feed speed, welding current, TCP y coordinate and equipment power draw showing synchronization between different measurement files for the third layer 3 of experiment WID 632 (PMC, CMT).

**Fig. 3:**
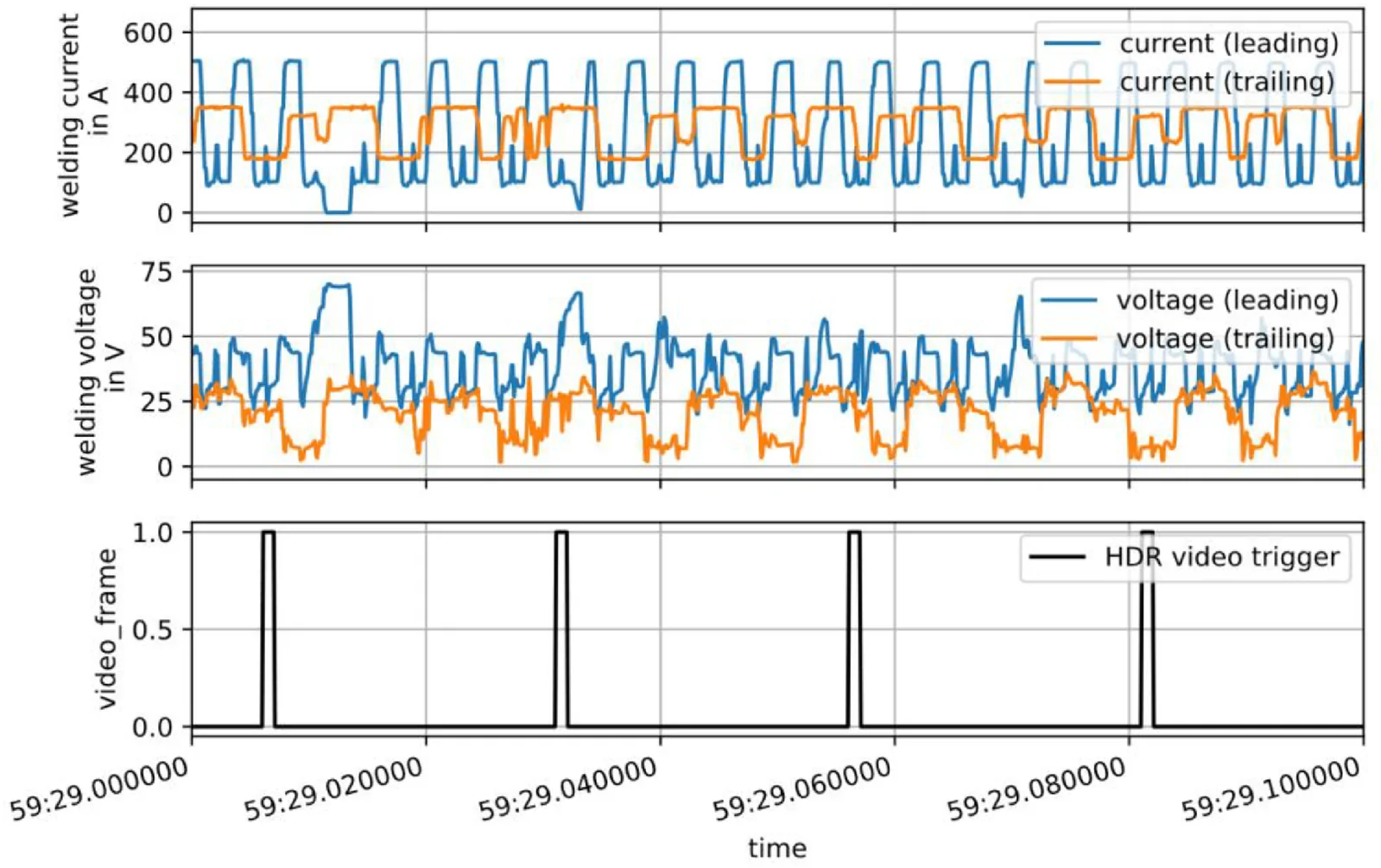
detailed section showing time series of measured welding current (top), welding voltage (middle) and HDR video frame capture (bottom) over a span of 0.1 s from the example shown in Fig. 2.

### 3.2 Surface scan data

For each recording, a 3D point cloud of the workpiece surface is recorded before welding and included in the file indicated by the suffix *_scan_pre.nc .* For the final 6th layer of each weld, an additional scan file suffixed*_scan_post.nc* is included in the dataset representing the final state of the workpiece after finishing the 6th layer. Hence, for layers 1–5 the 3D surface measured before welding the n ^th^ layer is found in the *_scan_pre.nc* file of the n^th^ weld and the resulting 3D surface after welding is represented in the *_scan_pre.nc* file of the (n + 1)^th^ weld. The full mapping is listed in Table 3. Additionally, the weld bead surface is cleared of slag using a metal brush before each scan. The 3D scan file data is stored as a HDF5 file in the NetCDF4 format. The surface scan files have three main dimensions *p, n, c* defining the data structure explained in Table 4. The scan files are obtained by recording individual line profiles along the groove as described in the Experimental Design section. The number of profile lines recorded is indexed by the variable *p*. The resulting point cloud data is stored in the *coordinates* variable and has the array shape *p*×*n*×*c.* To aid with visualization of the point cloud data, a simple triangulation is stored in the *triangles* variable. Fig. 4 shows examples overlaying the 800th profile in each scan for all seven scans collected for all experiments. The groove shape, workpiece thickness and layer by layer build up and bead surface shape is visible. In total the dataset consists of 47,188 recorded laser line profiles a 2048 recorded points across all T-GMAW weld layers.

**Table 3:** mappings between weld layer number and the filenames describing the measured 3D surface before and after the weld.

Weld layer number	3D surface before (filename suffix)	3D surface after (filename suffix)
1	_L1_T1_scan_pre.nc	_L2_T1_scan_pre.nc
2	_L2_T1_scan_pre.nc	_L3_T1_scan_pre.nc
3	_L3_T1_scan_pre.nc	_L4_T1_scan_pre.nc
4	_L4_T1_scan_pre.nc	_L5_T1_scan_pre.nc
5	_L5_T1_scan_pre.nc	_L6_T1_scan_pre.nc
6	_L6_T1_scan_pre.nc	_L6_T1_scan_post.nc

**Table 4:** description of data structure and variables in surface scan files.

variable name	type	unit	description
p	dimension	-	number of the recorded laser line profile
n	dimension	-	index of the recorded point along laser line
c	dimension	-	spatial dimension in welding reference frame
coordinates	data variable	mm	xyz-coordinates in the welding reference frame
triangles	data variable	-	simple triangulation indexes

**Fig. 4:**
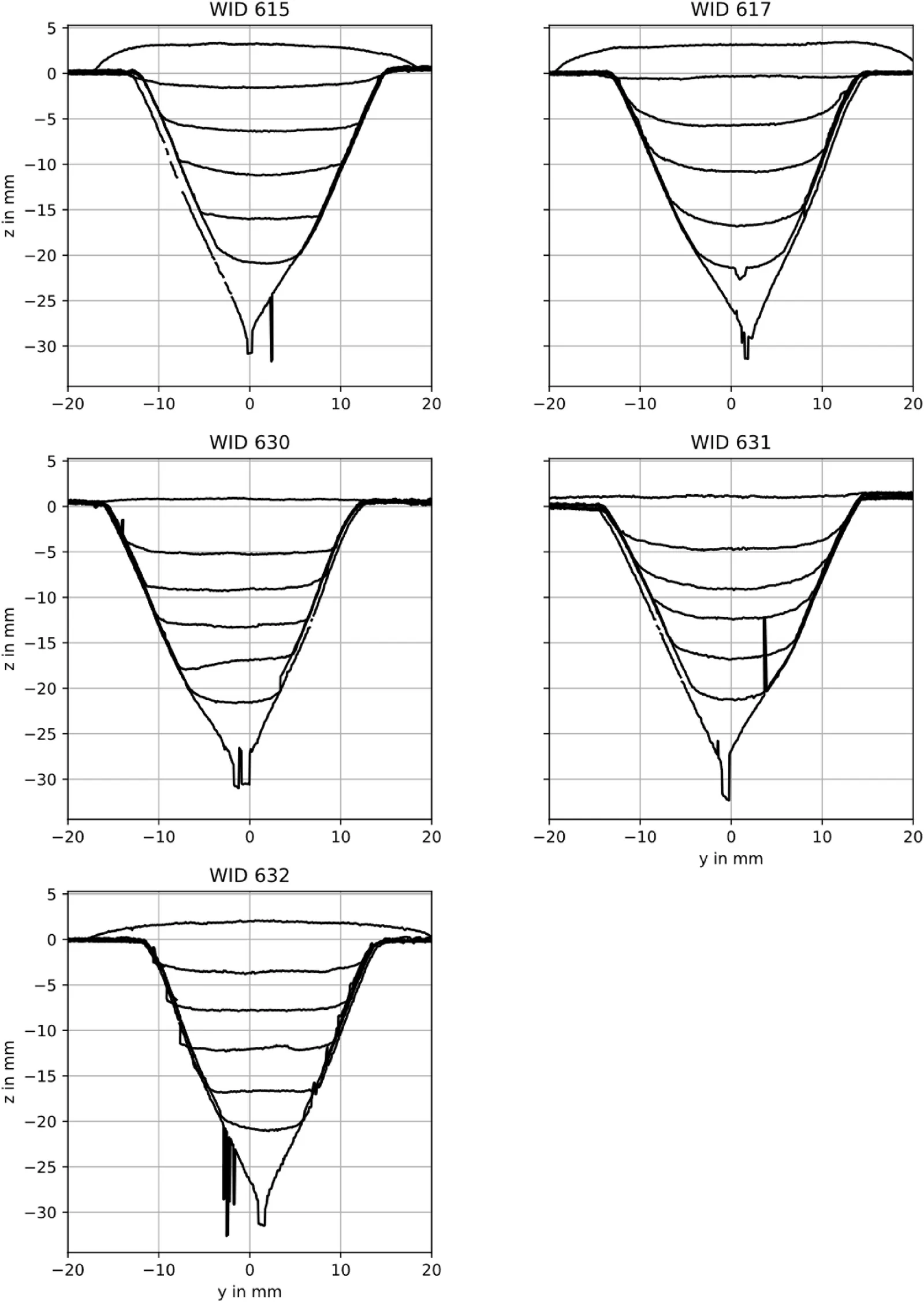
overlay plots of all seven scans at line 800 for all five experiments, with WID 632 corresponding to the TCP position of the measurement data shown in Fig. 3.

### 3.3 Video recordings

Each layer includes a monochrome high dynamic range (HDR) video file with the suffix *_video.avi* at a resolution of 1280 pixels × 1024 pixels. Video frames are individually triggered by a rising edge of the trigger signal *video_frame* (Table 2). The trigger signal is created at a frequency of 40 Hz by the real-time control system to ensure synchronization with the other time series data timestamps. The camera setup is described in the experimental design section. The dataset contains a total of 51,783 video frames recorded across all T-GMAW weld layers.

### 3.4 Data access and visualization example code

The dataset includes an IPython notebook file *data_view_example.ipynb* demonstrating how to read, plot and validate the different time series data and visualize the surface scan data together with the recorded movement of the welding process along the groove for each individual layer using Python.

## 4 Experimental Design, Materials and Methods

The welding experiments for data generation were conducted using a robotic arc welding setup at the welding laboratories of division 9.3 – Welding Technology at BAM. The setup consists of commercially available robotic and welding equipment with additional sensors for process monitoring. The welding torch was manipulated by a six-axis YASKAWA AR1730 robot operated via a YRC1000 controller (rated repeat accuracy 0.02 mm). The robot controller is equipped with an Ethernet/IP interface for communication with the welding equipment and EtherCAT interface for real-time communication with an external control system. The welding process is operated by two Fronius TPS-500i arc welding power sources, controlling the lead and trailing wires’ arc welding process respectively. Both power sources are linked with a proprietary high-speed interface to allow synchronisation between the leading and trailing wires welding processes. Each welding power source feeds its wire and welding current through a cable-hose of 10 m in length. The cable-hoses are joined at the T-GMAW welding torch (Fronius WF 60i TWIN Drive /W with Fronius MTB 2 × 500i). The leading and trailing wires are arranged at an 8° angle with a distance between the contact tips of 14 mm (Fig. 5).

**Fig. 5:**
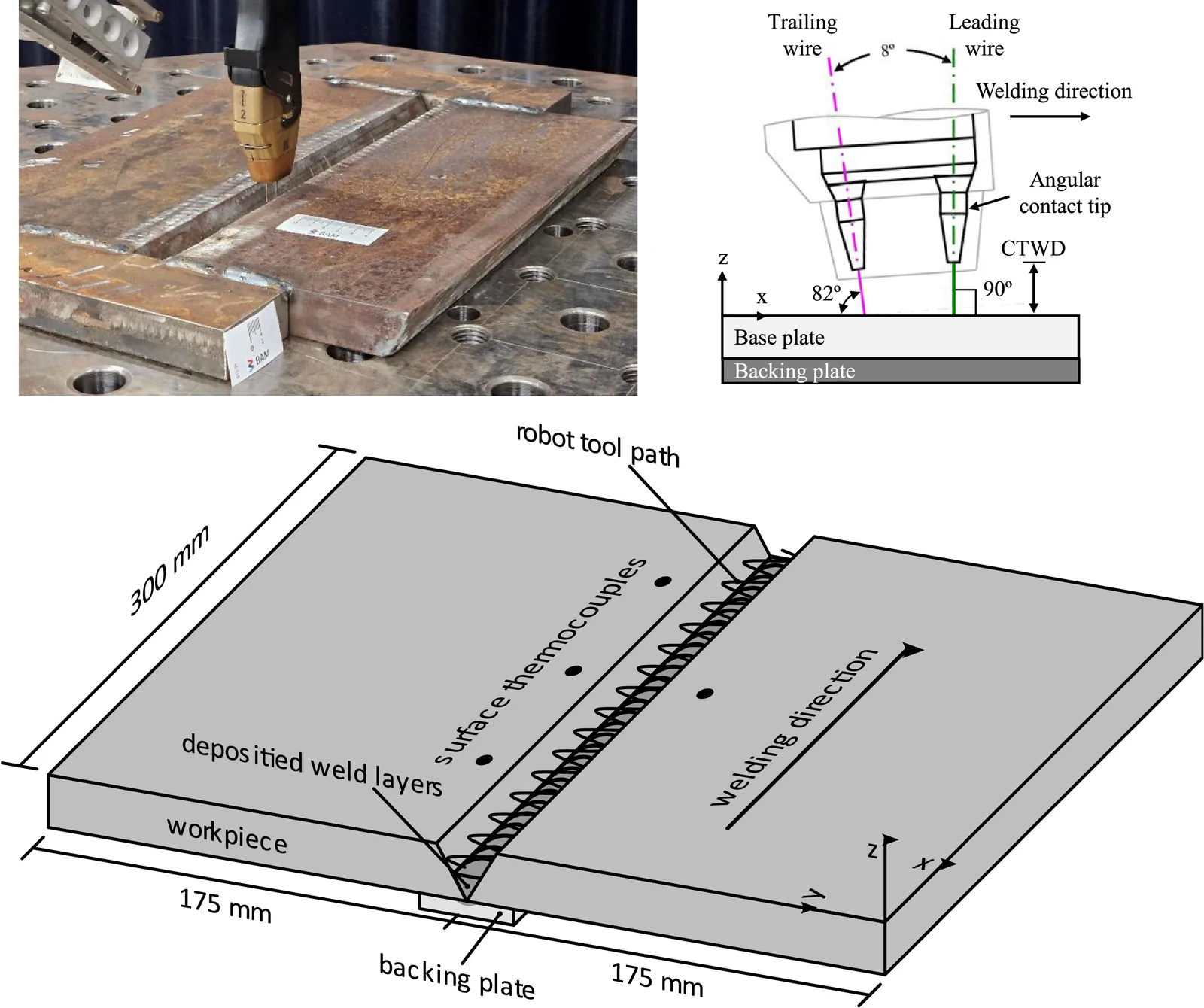
Photo of T-GMAW torch and workpiece without clamping (top, left), T-GMAW wire arrangement (top, right) [1], Workpiece dimensions, groove design and weaving bead welding strategy (bottom, not to scale).

Both the leading and trailing filler wires are of the same mild steel material ER70S-6 with a 1.2 mm wire diameter supplied from a spool. The wire composition is listed in Table 5. A shielding gas composition of 82% Ar and 18% CO2 (ISO14175:M21 - ArC-18) is supplied to the welding process zone via the T-GMAW torch. The nominal shielding gas flowrate is set to 20 l/min (for variables of recorded values see Table 2)

**Table 5:** Chemical composition (in wt.%) of base material and filler wire according to the manufacturer’s sheet.

	Fe	C	Si	Mn	Cr	Mo	Ni	P	S	Al	Co	Cu	W
**Base material** **(S355J2+N)**	Bal.	0.19	0.22	0.79	0.14	0.02	0.08	0.03	0.02	0.01	0.00	0.19	0.00
**Filler wire (ER70S-6)**	Bal.	0.08	0.95	1.64	0.03	0.00	0.02	0.01	0.01	0.00	-	0.01	-

The workpiece consists of two structural steel base plates of 30 mm thickness of grade S355J2+N according to DIN EN 10,025–2. The composition is listed in Table 5. For workpiece preparation, each sides’ plates are cut to at least 300 mm length and 175 mm width. The joint preparation is formed by flame cut of each base plate at a 25° angle along the long edge to form the resulting V-Groove with a 50° opening angle. The plates are manually tack welded together for positioning and keeping a root gap of roughly 1 mm to 2 mm along the groove. A steel backing plate with a ceramic backing plate inlay is used as a support. All welding is carried out in flat position (1 G).

### 4.1 Welding sequence and parameters

Since the 30 mm groove cannot be filled by a single weld pass, the same multi-layer welding strategy was used for all experiments. Welding was carried out over six layers, with each layer welded in a single-bead-per-layer strategy by adding a lateral sinusoidal weaving motion of the robot (Fig. 5). The chosen welding parameters of the individual experiments were selected based on the available literature and the capabilities of the available equipment. WID 617 was chosen as a baseline experiment to exactly follow the experiment described by Sproesser et al. in [4] regarding welding sequence, leading and trailing wire feed rates, welding speeds and weaving parameters. As such, WID 617 can be viewed as a repeat experiment of the existing literature with state-of-the-art T-GMAW equipment. Further, this experiment represents the maximum achievable deposition rate at combined wire feed rates up to 35 m/min. In comparison, WID 615 substitutes only the trailing wire welding process with CMT at the highest available wire feed rate of 12 m/min with the provided equipment. This experiment therefore can offer a good comparison to WID 617, achieving high deposition rates when combining PMC and CMT processes. Since 12 m/min wire feed rate is the maximum wire feed rate for CMT, the maximum combined wire feed rate for CMT+CMT is 24 m/min which is represented with WID 630. WID 631 and WID 632 offer the same wire feed rates at process combinations PMC+PMC and PMC+CMT respectively to allow a direct comparison at otherwise identical welding parameters. The amplitude and frequency of the weaving motion is adjusted based on the remaining groove width measured after the previous layer deposition. The weaving parameters are given in Table 6. Due to collision risks in the narrow groove angle by the weaving motion, the torch positioning is limited for the first two layers to a higher contact-tip-to-workpiece distance (CTWD) of 28 mm and 23.5 mm respectively. In addition, the initial root pass weld is carried out using only the leading wire in a single wire PMC process configuration to match the process configuration with previous results found in [4]. The workpiece was preheated to a temperature of around 100 °C before the initial root pass weld. The remaining weld passes were carried out with a CTWD of 20 mm measured for the leading wire (Fig. 5). Type-K thermocouples were tack welded to the workpiece surface in roughly 20 mm distance from the upper groove edge (Fig. 5) for monitoring the workpiece temperature and keeping an interlayer temperature of around 100 °C. For measurement of the t_8/5_ cooling time from 800 °C down to 500 °C, an attempt was made to manually place a Type-K thermocouple in the weld pool for each experiment. Due to the difficulty in positioning the thermocouple in this way, not all measurements were successful. Were possible, the t_8/5_ cooling time was manually extracted from the temperature recordings (see Table 1).

**Table 6:** Overview of process modes, set wire feed rate for the leading (L) and trailing (T) wires and combined (TO) wire feed rate, welding travel speed and welding path configuration; (M: Process mode, WFR: Wire feed rate, WTS: Welding travel speed, WV_a_: Weaving amplitude, WV_f_: Weaving frequency, CTWD: contact-tip-to-workpiece distance).

WID	Layer	M_L_	M_T_	WFR_L_	WFR_T_	WFR_TO_	WTS	WV_a_	WV_f_	CTWD
				m/min	m/min	m/min	mm/s	mm	Hz	mm
615	1	PMC	-	12.5	-	12.5	6.67	0.65	2.5	28
	2	PMC	CMT	15	12	27	10	1.5	1.4	23.5
	3	PMC	CMT	20	12	32	7	5	0.8	20
	4	PMC	CMT	20	12	32	6.33	7.5	0.6	20
	5	PMC	CMT	20	12	32	5.33	10	0.5	20
	6	PMC	CMT	12.5	8	20.5	3	11.75	0.33	20
617	1	PMC	-	12.5	-	12.5	6.67	0.65	2.5	28
	2	PMC	PMC	15	12.5	27.5	10	1.5	1.4	23.5
	3	PMC	PMC	20	15	35	7	5.5	0.8	20
	4	PMC	PMC	20	15	35	6.33	8	0.6	20
	5	PMC	PMC	20	15	35	5.33	10	0.5	20
	6	PMC	PMC	12.5	8	20.5	3	11.75	0.3	20
630	1	PMC	-	12	-	12	6.67	0.65	2.5	28
	2	CMT	CMT	12	12	24	10	4.5	1.4	23.5
	3	CMT	CMT	12	12	24	7	6.5	1	20
	4	CMT	CMT	12	12	24	6.33	8.9	1	20
	5	CMT	CMT	12	12	24	5.33	11	0.7	20
	6	CMT	CMT	12	12	24	3	12.25	0.8	20
631	1	PMC	-	12	-	12	6.67	0.65	2.5	28
	2	PMC	PMC	12	12	24	10	4.5	1.4	23.5
	3	PMC	PMC	12	12	24	7	6.5	1	20
	4	PMC	PMC	12	12	24	6.33	8.9	1	20
	5	PMC	PMC	12	12	24	5.33	11	0.7	20
	6	PMC	PMC	12	12	24	3	12.25	0.8	20
632	1	PMC	-	12	-	12	6.67	0.65	2.5	28
	2	PMC	CMT	12	12	24	10	4.5	1.4	23.5
	3	PMC	CMT	12	12	24	7	6.5	1	20
	4	PMC	CMT	12	12	24	6.33	8.9	1	20
	5	PMC	CMT	12	12	24	5.33	11	0.7	20
	6	PMC	CMT	12	12	24	3	12.25	0.8	20

### 4.2 Measurement setup

The measurement setup is built around a PC-based real time control system. The control system is operated by the TwinCAT3 Runtime at a base task cycle time of 0.1 ms. Recording triggers and measurements are driven by fieldbus devices connected via an EtherCAT fieldbus operated by the TwinCAT3 Runtime with the distributed clocks functionality for precise timing between signals. Time series recording is implemented via the TwinCAT3 Scope Server software at the sampling intervals per channel listed in Table 2. Fig. 6 shows an overview of the control system and measurement layout which has previously been described in more detail in [7]. For measuring the welding currents, HKS P700/1000 compensated Hall sensors (rated accuracy < ± 1%) are placed along each supply cable. For welding voltage measurement, the sensors are connected at the power source terminal and workpiece respectively. The P700/1000 sensors convert the measurements to a −10 V to +10 V range which is measured using Beckhoff ELM3002–0030 (rated accuracy < ± 0.011%) analog to digital conversion terminals. The terminal values are collected via the EtherCAT fieldbus in the control system. The instantaneous power draw of each power source and the robot system are measured at the respective wall plug connectors using 50 A / 1 A current transformers (MBS ASRD 14, rated accuracy < ± 1 %) and Beckhoff EL3443–0020 (rated accuracy < ± 0.6%) power measurement terminals integrated into the EtherCAT fieldbus (Fig. 7 and Table 2). The shielding gas flow rate supplied to the process for each wire is measured at the gas bottle supply line using a Bronkhorst EL-Flow Select gas flow controller which is integrated into the EtherCAT field bus. Furthermore, gas flow rates are also reported by each power source via OPC-UA protocol (Fig. 6 and Table 2). Since all time series measurements and video triggers are collected and recorded via the real-time TwinCAT 3 Runtime, all recorded timestamps — even across multiple files — reference the same clock operating the EtherCAT master. The EtherCAT master has distributed clocks enabled for the analog-to-digital Beckhoff EL terminals to ensure the synchronization drift between collected signals to be less 10 µs.

**Fig. 6:**
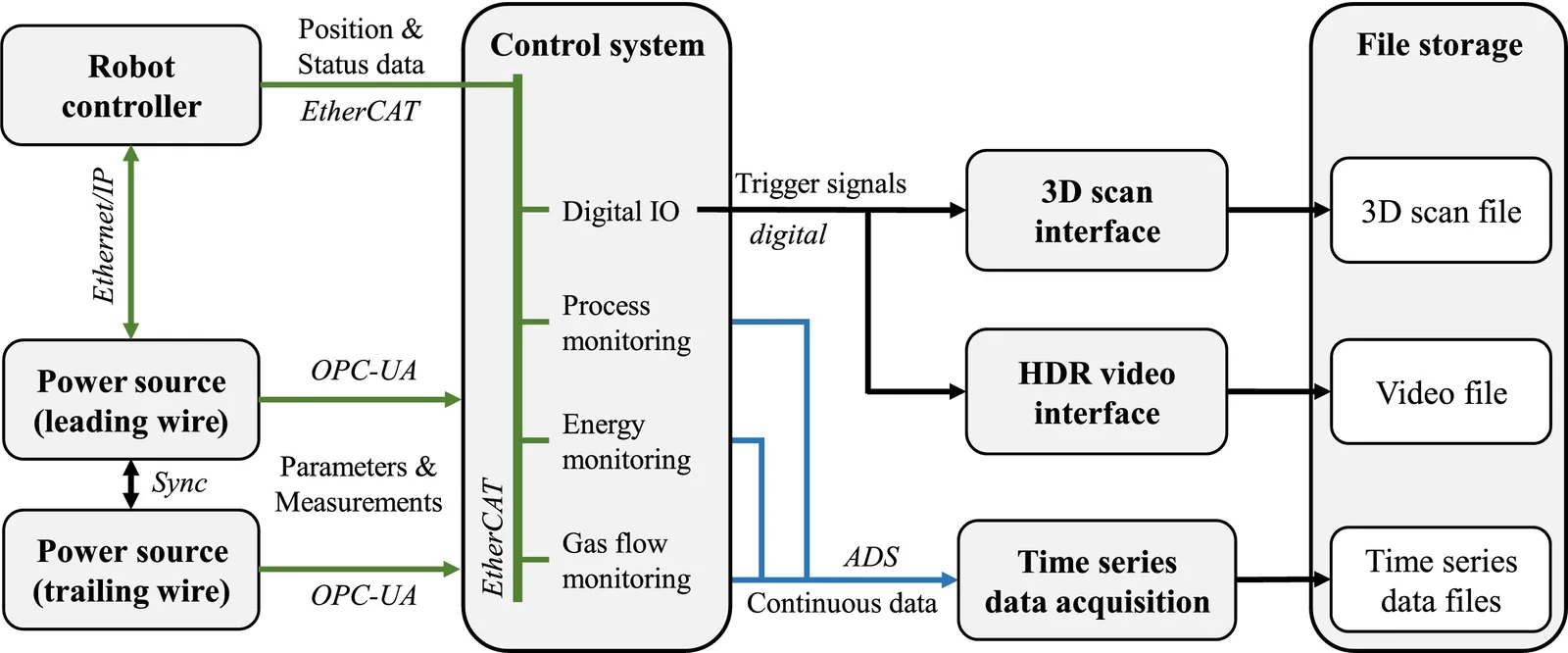
Control system layout and dataset generation, adopted for T-GMAW setup from [7].

**Fig. 7:**
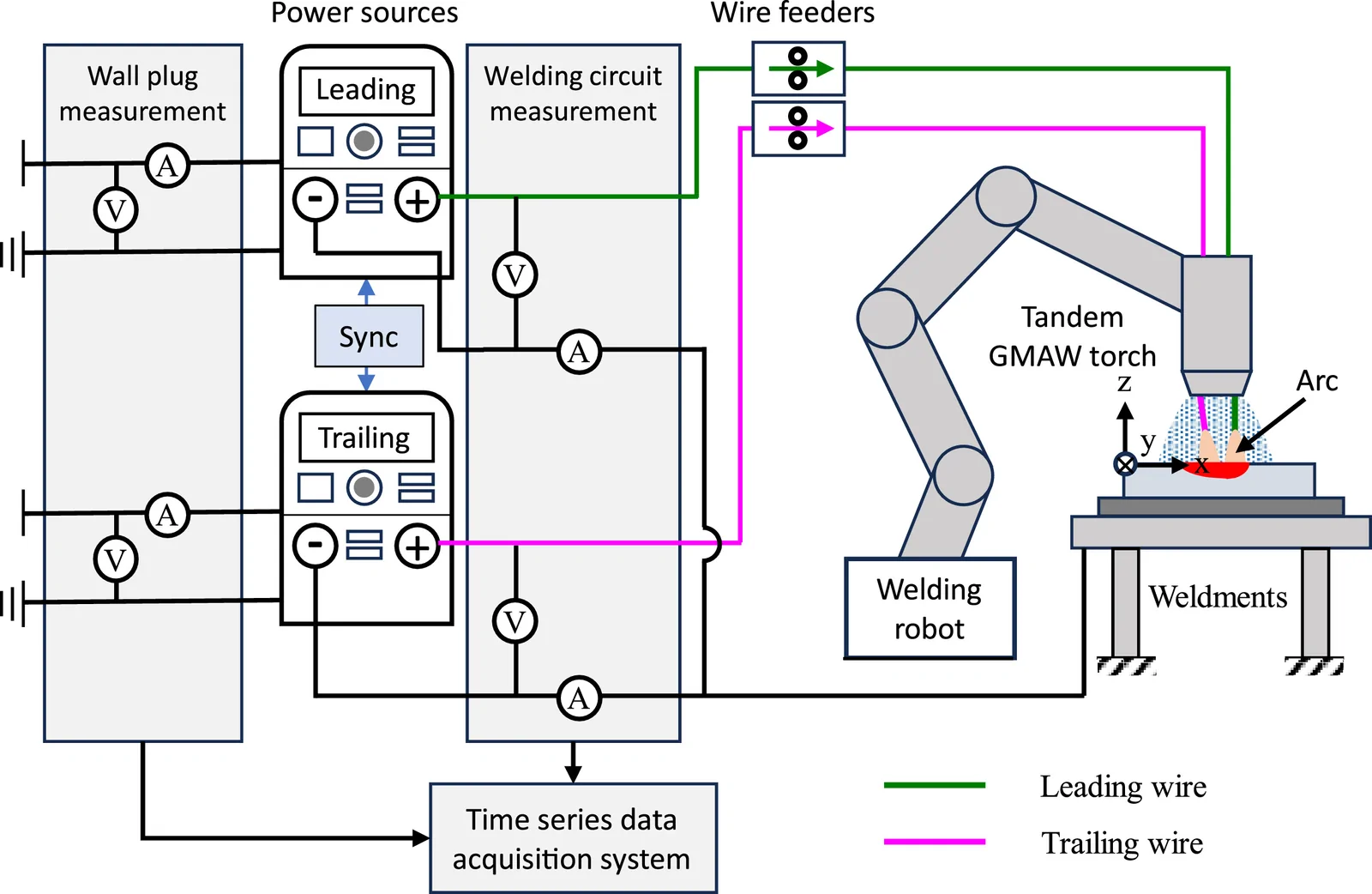
Welding connectivity and system layout for power draw and welding process signatures measurement, expanded from [1].

The 3D surface data is recorded by a laser line triangulation sensor (Micro-Epsilon scanCONTROL 3010–100, rated accuracy ± 0006%) that is mounted on the robot. Before the welding of each layer, the robot is moving the sensor along the weld seam with the laser line projecting perpendicular to the weld seam and welding direction. During the movement, individual laser line measurements get triggered via digital input signals from the real time control system at a rate of 50 Hz. The sensor records 2048 points along a laser line length of around 100 mm, resulting in a lateral resolution of roughly 0.05 mm. For each laser line recorded, the robot position is stored alongside the triangulation data. This allows the data to be transformed into the welding reference frame by the software interface. After the final deposition of the 6th layer, one additional surface measurement is taken representing the state of the completed weld.

For video recording of the welding process and melt pool dynamics, a monochrome high dynamic range welding camera (Xiris XVC-1000) with a 35 mm lens (Ricoh FL-CC3516–2 M), 5 mm CS to C-Mount spacer and a UV protective filter is mounted behind the welding torch looking in welding direction to show a rear view of the process and melt pool dimension at a roughly 45° inclination and 350 mm distance. The camera is operating in global shutter mode at a fixed exposure time of 2 ms. Video recording is managed with the Xiris WeldStudio3 software without any additional image processing enabled.

## Limitations

The data provided is limited to the combination of S355J2+N base material with ER70S-6 filler material in flat position as mentioned. No other material combinations or welding positions are investigated. All experiments have been conducted with the described Fronius power sources and welding equipment. As such, results for welding processes provided by other manufacturers with similar arc welding processes compared to PMC and CMT operating modes might yield different results depending on the proprietary process control.

The initial root pass is welded using a single wire PMC process for all experiments using the T-GMAW equipment. As such, the dataset does not cover root pass welding using T-GMAW.

The selected welding experiments and process parameter combinations cover only a selected area of the total parameter space available to T-GMAW applications.

The t_8/5_ cooling times are not available for the following experiments due to insufficient attachment during the manual positioning placement in the weld pool: WID 630 – Layer 6, WID631 Layers 5 & 6, WID 632 – Layers 2 & 6.

The included video data is provided without intrinsic or extrinsic camera calibration parameters.

## Ethics Statement

The current work does not involve human subjects, animal experiments, or any data collected from social media platforms. The authors have read and followed the ethical requirements for publication in Data in Brief.

## CRediT Author Statement

**Çağtay Fabry**: Writing - Original Draft, Writing - Review & Editing, Conceptualization, Investigation, Data Curation, Software, Validation. **Prashant Kumar Chaurasia:** Writing - Review & Editing, Conceptualization, Investigation. **Andreas Pittner:** Writing - Review & Editing, Conceptualization**,** Funding acquisition, **Michael Rethmeier:** Writing - Review & Editing, Conceptualization**,** Funding acquisition

## References

[1] P.K. Chaurasia, Ç. Fabry, A. Pittner, A. De, M. Rethmeier, Tandem gas metal arc welding of thick steel plates: progress in productivity and properties using advanced process modes, Sci. Technol. Weld. Join. (2026) doi:10.1177/13621718261472548.

[2] Chaurasia P.K., Fabry Ç., Pittner A., Rethmeier M. (2026). Tandem-GMAW: advanced pulsed and short-circuiting process mode experimental data. Zenodo.

[3] Sproesser G., Chang Y.-J., Pittner A., Finkbeiner M., Rethmeier M. (2015). Life cycle assessment of welding technologies for thick metal plate welds. J. Clean. Prod..

[4] Sproesser G., Pittner A., Rethmeier M. (2016). Increasing performance and energy efficiency of gas metal arc welding by a high power tandem process. Procedia CIRP.

[5] Ueyama T., Ohnawa T., Tanaka M., Nakata K. (2005). Effects of torch configuration and welding current on weld bead formation in high speed tandem pulsed gas metal arc welding of steel sheets. Sci. Technol. Weld. Join..

[6] Liu G., Han S., Tang X., Cui H. (2021). Effects of torch configuration on arc interaction behaviors and weld defect formation mechanism in tandem pulsed GMAW. J. Manuf. Process..

[7] Fabry Ç., Pittner A., Kaplan A., Rethmeier M. (2026). Digitalisation and carbon footprint evaluation of gas metal arc welding and wire-arc directed energy deposition. IOP Conf. Ser.: Mater. Sci. Eng..

